# Professional Preferences Towards Vaginal Breech Delivery at Term: An International Discrete Choice Experiment

**DOI:** 10.1111/birt.70011

**Published:** 2025-09-01

**Authors:** Merle R. van Dijk, Lotte van Wijk, Leonie E. Van Rheenen‐Flach, Etelka Moll, Wessel Ganzevoort, Joost Velzel

**Affiliations:** ^1^ Department of Obstetrics and Gynecology OLVG Amsterdam North‐Holland the Netherlands; ^2^ Department of Obstetrics and Gynecology Amsterdam University Medical Centers Amsterdam North‐Holland the Netherlands; ^3^ Department of Obstetrics and Gynecology Northwest Clinics Alkmaar North‐Holland the Netherlands

**Keywords:** breech deliveries, cesarean delivery, counseling, vaginal breech delivery

## Abstract

**Objective:**

To assess healthcare professionals' preferences for antepartum counseling on the mode of delivery for patients with term breech presentation and their preferences for intrapartum management of vaginal breech delivery (VBD).

**Study Design:**

A web‐based international survey and Discrete Choice Experiment (DCE) were conducted among gynecologists and midwives. In the DCE for antepartum counseling, participants were presented with scenarios for antepartum counseling and asked to choose between VBD and elective cesarean delivery (CD). The scenarios differed in four attributes: maternal age, BMI, parity, and estimated fetal weight. Intrapartum management of VBD was evaluated through a questionnaire involving four distinct clinical scenarios.

**Results:**

In antepartum counseling, the likelihood of being advised for a VBD was 43.9%. The strongest factors to opt for a CD compared to a VBD were a history of vaginal birth (OR 0.25, 95% CI 0.19–0.32) and a large estimated fetal weight (OR 5.89, 95% CI 4.07–8.54). Intrapartum management during breech deliveries did not differ among professionals and their annual number of self‐reported VBD.

**Conclusions:**

The key factors driving professionals to recommend CD are a history of vaginal birth and average estimated fetal weight. Intrapartum management approaches during breech deliveries were consistent across professionals. Those with more experience managing VBDs were more likely to counsel patients towards VBD antepartum and proceed with VBD intrapartum.

## Graphical abstract

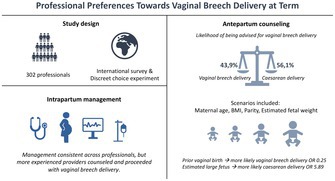

## 1 Introduction

Breech presentation continues to present a clinical dilemma regarding the mode of delivery [1]. While planned cesarean delivery (CD) benefits neonatal outcomes, planned vaginal breech delivery (VBD) offers better maternal outcomes, as CD is associated with increased risks of short‐ and long‐term reproductive health complications [2, 3]. The Term Breech Trial (TBT) demonstrated that elective CD reduces neonatal mortality and severe morbidity (relative risk 0.33 [95% CI 0.19–0.56]), leading to its widespread adoption as the preferred delivery method [4, 5, 6]. However, the TBT has faced criticism, and not all subsequent studies have confirmed its findings [7, 8, 9, 10]. A meta‐analysis by Berhan et al. reported that the risk of neonatal mortality is four times higher with VBD compared to elective CD, though the absolute risk remains low, requiring a high number of CDs (up to 1:338) to prevent a single case of neonatal mortality or morbidity [2, 11].

Despite the maternal and reproductive disadvantages of elective CD, VBD should remain an option for patients, particularly since the relevant risks of VBD may be attenuated in the hands of experienced and skilled obstetric caregivers [7, 12, 13]. In the Netherlands, VBD rates stabilized at 35% from 2015 to 2019, indicating that many patients still view VBD as a reasonable choice [14]. To ensure the safety of VBD, certain relative antepartum and intrapartum contraindications have been recognized in the literature, although high‐quality evidence for these is lacking [7, 15]. Additionally, counseling plays a crucial role, with the counselor's perspective significantly influencing the patient's decision [16].

Given these factors, there is considerable international variation in practice, often leading to the overuse of elective and emergency CD [2, 17]. To better understand the variation in clinicians' perspectives, we conducted an international questionnaire study. The primary aim was to identify contributing factors influencing obstetric caregivers and breech experts when counseling patients with term breech presentations on their mode of delivery. Secondary objectives were to assess whether the self‐reported experience of the caregiver impacts counseling and to examine differences in intrapartum management based on caregiver experience.

## 2 Materials & Methods

A web‐based questionnaire was distributed to obstetric departments in the Netherlands and to gynecologists and midwives with breech expertise in other countries. The questionnaire included three sections: participant baseline characteristics, a Discrete Choice Experiment (DCE) on antepartum counseling, and a survey on intrapartum management. Participants were asked to forward the survey to colleagues with similar expertise. The survey link was emailed in May 2020 via Qualtrics XM (Provo, UT), with a reminder sent after one week to non‐responders. The survey remained open until October 4, 2020. As the study did not involve patients, ethical approval was not required. The ISPOR Guidelines and a DCE checklist were followed [18, 19]. All scenarios are available in Appendix S1.

### 2.1 DCE for Antepartum Counseling

In the DCE, participants were randomly assigned six hypothetical scenarios, choosing between planned VBD or elective CD for each as the best option for their patient, defined by different attributes and varying levels to explore decision‐making factors. This method identifies which of the attributes are influential in the decision making [20]. Each scenario described a well‐descended single fetus in frank breech presentation, with an adequate pelvis (in case of uncertainty, determined with MR pelvimetry) and a patient with a neutral opinion between planned VBD or elective CD [21]. A literature search was performed to determine relevant factors in counseling patients in singleton breech pregnancies near term [15, 22]. Four attributes and two or three corresponding levels were extracted from literature and discussed in the panel of experts [23]. The outcomes of interest consisted of: maternal age (22, 32 or 42 years), BMI (23 or 32), parity (nulliparity, multipara with in the obstetric history a normal vaginal birth and multipara with in the obstetric history a cesarean for dystocia) and the estimated fetal weight (2200, 3200 or 4100 g).

### 2.2 Survey for Intrapartum Management

The final part consisted of multiple‐choice questions regarding intrapartum management during the VBD of a singleton, frank breech presentation at term. Participants were asked about their management approach in four scenarios involving either 15 or 30 min of fetal distress (based on an abnormal cardiotocography (CTG) monitoring) during active labor with a breech delivery at Hodge 3 or 4 (where Hodge 3 represents descent to the mid‐pelvis, and Hodge 4 indicates descent to the pelvic outlet, nearly crowing) [24]. Participants were asked to choose one of the following management options: continue pushing, breech extraction, emergency CD, or other.

### 2.3 Sample Size and Statistical Analysis

Based on DCE sample size requirements, 125 participants were needed, assuming six choice tasks, two alternatives, and up to three attribute levels [25, 26].

Baseline characteristics and survey outcomes were analyzed descriptively, reporting categorical variables as frequencies and percentages, and continuous variables as means with standard deviations and minimum and maximum values. Incomplete responses were excluded from further analysis.

For the primary analysis, first a univariable analysis was performed to evaluate the association between individual attributes and the choice of delivery mode. Subsequently, statistically significant factors identified in the univariable analysis were then included in a multivariable analysis to assess their associations with the outcome while adjusting for the other factors. A Generalized Estimating Equations (GEE) model was used in both the univariable and multivariable analyses to account for repeated measures on the same participant, given that each participant evaluated multiple scenarios. The GEE model was specified with an exchangeable correlation structure to manage within‐subject variability, allowing for robust estimation of associations between attributes and the likelihood of selecting a particular mode of delivery. Odds ratios (OR) and 95% confidence intervals (CI) were reported to convey the strength and precision of these associations. Secondary analysis examined if clinician experience affected the counseling, dividing participants into quartiles based on the number of self‐reported breech deliveries in the past five years. We extended the univariable GEE models with an interaction term for individual attributes with quartile of clinical experience and used the overall Wald Chi square test for the interaction term to determine statistical significance of the effect of clinician experience. The intrapartum management based on caregiver experience was analyzed descriptively.

Statistical significance was set at a *p*‐value < 0.05 for all analyses. Analyses were conducted using IBM SPSS Statistics (Version 29.0, Armonk, NY: IBM Corp).

## 3 Results

In total, 302 professionals completed the questionnaire of whom 89.4% were gynecologists. Most responders (91.4%) were based in Europe (91.4%). The average clinical experience was 15.5 years, and the mean number of breech deliveries in the last five years was 20.2. Table 1 shows the baseline characteristics of all respondents.

**TABLE 1:** Baseline characteristics (*n* = 302).

	*n* (%)		
Profession			
Gynecologist	89.4%		
Midwife	5.3%		
Other	5.3%		
Continent			
Australia	0.3%		
Europe	91.4%		
North America	1.7%		
South America	2.3%		
South Africa	3.6%		

	Mean	Minimum	Maximum
Clinical experience (years)	15.5	1	45
Number of breech deliveries in last five years	20.2	0	350

*Note:* Baseline characteristics of the total cohort.

Abbreviation: *n* = number.

### 3.1 Antepartum Counseling

A total of 1.812 scenarios were evaluated, and each scenario was assessed by a minimum of 33 professionals. VBD was selected in 43.9% of the cases, while an elective CD was chosen in 56.1%.

Table 2 summarizes the attributes and levels assessed in the DCE, as well as the associations between antepartum characteristics and the selection of CD. Univariable analysis revealed that all predetermined characteristics significantly influenced the chosen mode of delivery. CD was more frequently chosen for older patients, those with a higher BMI, or those with large‐for‐gestational‐age (LGA) fetuses estimated fetal weight (EFW) (*p* < 90). In multivariable analysis, again all characteristics were associated with the choice for mode of delivery. The strongest predictors for CD were multiparity with a history of cesarean delivery for dystocia (OR 5.32 95% CI 3.84–7.37) and an EFW *p* > 90 (OR 5.89, 95% CI 4.07–8.54).

**TABLE 2:** Univariable and multivariable analyses of antepartum characteristics and opting for VBD.

		Univariable analysis				Multivariable analysis opting for CD	
Attribute	Level	VBD (%)	Elective CD (%)	OR (95% CI)	*p*	OR (95% CI)	*p*
Age	22 years	49.7%	50.3%		< 0.001	^a^	< 0.001
	32 years	44.9%	55.1%	1.21 (0.99–1.48)		1.29 (0.99–1.69)	
	42 years	37.2%	62.8%	1.67 (1.33–2.10)		2.04 (1.53–2.73)	
BMI	Normal (23)	46.9%	53.1%		0.014	^a^	0.007
	High (32)	41.0%	59.0%	1.27 (1.05–1.54)		1.34 (1.09–1.73)	
Parity	Nullipara	44.9%	55.1%		< 0.001	^a^	< 0.001
	Multipara (in obstetric history normal vaginal birth)	71.0%	29.0%	0.33 (0.26–0.42)		0.25 (0.19–0.32)	
	Multipara (a cesarean in obstetric history for dystocia)	16.0%	84.0%	4.3 (3.20–5.78)		5.32 (3.84–7.37)	
Estimated fetal weight	SGA (< p10)^b^	50.0%	50.0%			^a^	< 0.001
	Average (p50)	60.7%	39.3%	0.65 (0.49–0.85) 3.82		0.55 (0.39–0.77)	
	LGA (> p90)^c^	20.7%	79.3%	(2.87–5.09)		5.89 (4.07–8.54)	

*Note:* The a priori chance to opt for VBD is 43.9%. The univariable analysis shows how each individual level does or does not affect the decision‐making in the outpatient clinic.

^a^ The multivariable analysis shows the levels and their corresponding odds ratio, indicating how each level increases the a priori chance (43.9%) for obstetricians to opt for an elective CD, in comparison with the reference category (a VBD). Good fit to data (*p* < 0.001).

^b^ SGA = small for gestational age.

^c^ LGA = large for gestational age.

The association between characteristics and the choice of CD across quartiles (Q) based on the number of self‐reported breech deliveries in the last five years was analyzed. Participants in Q1 assisted 0–8 breech deliveries, Q2 assisted 9–15, Q3 assisted 16–25, and Q4 assisted 26–350. The likelihood to opt for VBD was 36.1% in Q1, 42.9% in Q2, 50.3% in Q3, and 50.9% in Q4. In comparing quartile groups Q1–Q4, none of the assessed determinants reached statistical significance when considering the overarching *p*‐values. Specifically, the *p*‐values were as follows: age (*p* = 0.679), BMI (*p* = 0.304), parity (*p* = 0.512), and estimated fetal weight (*p* = 0.379). These results suggest no statistically significant differences across the quartiles for these characteristics in terms of their influence on the selected mode of delivery.

### 3.2 Intrapartum Management

Regarding intrapartum management for breech deliveries at term, 47.4% of respondents indicated they would permit induction in all cases, including cervical priming. Induction with a favorable cervix was allowed by 22.5%. Augmentation of labor, by administration of oxytocin, for inadequate contractions or lack of progress would be performed by 74.5% and 63.2%, respectively. During labor, the majority of professionals required continuous cardiotocography (CTG) monitoring (81.8%), allowed pain relief (94.7%), position changes (95.0%), and upright VBD (58.6%). Additionally, 87.4% reported immediate access to emergency CD, and 89.1% had a pediatrician on standby.

Table 3 presents the results of various management strategies for fetal distress during active labor. Professionals with less VBD experience more frequently reported performing an emergency CD for a 15 min abnormal CTG at Hodge 3 (78.6% in Q1 vs. 64.2% in Q4). Fewer experienced professionals continued pushing with abnormal CTG for 30 min at Hodge 3 (8.3% in Q1 vs. 1.9% in Q4).

**TABLE 3:** Management of fetal distress during active labor.

	Breech descended in pelvis	Duration abnormal CTG^a^		Continue active pushing	Breech extraction	Emergency cesarean delivery	Other
1	Hodge 3/Zero station	15 min	Total cohort (*N* = 302)	11.0%	5.0%	67.1%	16.9%
			Q1	8.3%	7.1%	78.6%	6.0%
			Q4	7.5%	3.8%	64.2%	24.5%
2	Hodge 3/Zero station	30 min	Total cohort (*N* = 302)	4.7%	5.7%	80.9%	8.7%
			Q1	8.3%	3.6%	85.7%	2.4%
			Q4	1.9%	5.8%	84.6%	7.7%
3	Hodge 4/Crowning	15 min	Total cohort (*N* = 302)	48.5%	31.8%	10.0%	9.7%
			Q1	51.2%	32.1%	11.9%	4.8%
			Q4	50.9%	30.2%	9.4%	9.4%
4	Hodge 4/Crowning	30 min	Total cohort (*N* = 302)	14.7%	49.3%	20.7%	15.3%
			Q1	11.9%	53.6%	25.0%	9.5%
			Q4	11.5%	57.7%	21.2%	9.6%

*Note:* Management strategies of fetal distress during active labor in several different scenarios. Presented for the total cohort as well as the subdivision in Q1 and Q4, according to the self‐reported number of breech deliveries assisted in the last five years.

^a^ Cardiotocography.

## 4 Discussion

### 4.1 Main Findings

This international cohort of professionals guiding breech birth demonstrated that VBD is widely available within this cohort, and professionals guiding more patients annually on VBD are more likely to counsel towards VBD antepartum and proceed with VBD intrapartum. In the antepartum setting, the most influential factor for professionals to counsel towards VBD is a vaginal birth in obstetric history. For intrapartum management, the majority of the participating obstetricians allowed induction and augmentation of labor, required continuous CTG monitoring, allowed pain relief, and changing of position. Fetal distress during active labor was treated variably and was influenced by the descent of the breech in the pelvis and the duration of an abnormal CTG.

### 4.2 Strengths and Limitations

To our knowledge, this study is the first study using a DCE to examine professionals' preferences towards VBD in the case of singleton term breech presentation. By following a clear methodology based on guidelines, we obtained a representative reflection of international daily practice on breech delivery management. Our study has limitations as well. First, we were restricted to a limited number of attributes and levels. Nevertheless, the chosen characteristics are easily available, clinically relevant, and well supported by literature [15, 22]. Breech birth management is inherently complex, and a key limitation of this study is that real‐world decision‐making cannot be fully captured in the simplified scenarios we analyzed. In clinical practice, additional factors and contextual nuances contribute to decision‐making, which vary according to the individual experiences, hospital protocols, and personal preferences of each healthcare provider. Each respondent may interpret and apply these scenarios in their own way, influenced by prior training and institutional guidelines. Nonetheless, this study stresses several critical factors, particularly highlighting the influence of clinical experience on management approaches.

In this study, we selected distinct groupings for characteristics within our scenarios to better identify factors that contribute to the decision to opt for cesarean delivery (CD). By using pronounced differences between attributes like age and BMI, we aimed to isolate the impact of these factors on delivery mode choices more clearly. In clinical practice, however, where patient profiles may present with more moderate variations, the influence of these specific factors on CD recommendations might be less pronounced. In addition, our online survey did not capture other non‐clinical determinants, such as hospital type, delivery volume, cesarean section rate, or neonatal outcomes, that could potentially influence both DCE choices and intrapartum management. We recognize that these and other factors are critical in shaping counseling, intrapartum management, and the outcomes of breech births.

A further limitation of this study is the reliance on self‐reported data regarding the number of breech births attended in recent years. These estimates may vary, and certain management choices by self‐identified experts might not align with standard protocol‐based practices valued by other practitioners. These discrepancies may arise due to variations in protocols, hospital practices, and regional experience across different healthcare systems.

We observed significant variation in intrapartum management decisions, closely tied to the practitioners' self‐reported experience with breech deliveries. Experienced practitioners likely have greater confidence and skill in managing challenging vaginal breech births, especially in discerning when intervention is necessary to ensure safe delivery. However, their heightened awareness of associated risks likely makes them more attuned to the importance of reverting to cesarean delivery when warranted, a skill essential for safeguarding maternal and neonatal outcomes in high‐risk situations. A final limitation is the open nature of our study, a method chosen to reach as many professionals as possible. This likely resulted in a bias of participants more likely to favor VBD, an inherent bias of survey studies. However, we think it is unlikely that the direction of our findings regarding decision factors within VBD would have changed if this study had recruited more respondents with a preference towards CD.

### 4.3 Interpretations

The most important questions in counseling patients on VBD are the chance of a successful VBD and the chance of poor neonatal outcome, both justifications for elective CD. Unbiased prediction models for these outcomes are lacking. However, risk factors for VBD resulting in emergency CD and poor neonatal outcome are available in literature [4, 11]. For antepartum counseling, we derived four attributes with two or three levels from literature, and we anticipated a gradual increase in the chance for elective CD per level. Our study clearly showed that counseling towards VBD is less likely when these risk factors are present in the scenario, such as nulliparity and higher estimated fetal weight. Therefore, the findings of our study help to understand the quantified influence of known risk factors in obstetricians' perceptions. Even though in this study quantification is based on qualitative research, the results provide valuable insights to support subsequent large studies on VBD as this study had sufficient power. The most likely scenario in which professionals opt for VBD was multiparity with a vaginal birth in obstetric history, which corresponds to findings of other studies indicating a higher chance of successful VBD in multiparity [15, 27, 28]. Our finding that LGA increases the chance to be counseled for CD compared to VBD (OR 5.89, 95% Cl 4.07–8.54) contradicts studies indicating that neonates with an estimated birth weight of > 4000 g are not at increased risk for morbidity and mortality after a VBD [5, 29]. Our study focused on professional preferences, whereas in the context of counseling in breech delivery, the motives of pregnant patients are likely most important. Little is known about their motives, and future research should focus on this. This study emphasizes the significant impact that healthcare providers have on counseling for breech birth. While our focus was on individual practitioners, examining team‐based decision‐making within healthcare teams would also be valuable. Decisions made collaboratively as a team, or those formalized in a protocol, may better support less experienced providers by reducing the influence of individual biases or past experiences.

Our finding that professionals who guide more patients annually on VBD tend to have a more positive attitude towards VBD suggests that centralizing care for counseling and performing VBD could be beneficial. Centralization of high‐complexity and low‐volume care has shown benefits in numerous situations [30]. In the case of breech management at term, improved outcomes and reduced healthcare costs can be expected from centralized care. A recently published Dutch retrospective cohort study in a single center dedicated to VBD demonstrated a high success rate of VBD with favorable neonatal outcomes [7, 17].

Alongside centralization, a structured, collegial approach within hospitals could enhance consistency in breech management. A collegial model, where less experienced obstetricians are supported by more experienced colleagues, with protocols for eligibility, labor induction, and standardized intrapartum practices, could provide similar benefits in environments where full centralization may not be feasible. Such protocols ensure that patient selection, management strategies, and readiness for emergency cesarean are uniformly applied, potentially achieving similar outcomes to centralized care. Centralization of breech management or the establishment of specialized clinics could still promote freedom of choice for patients, but these settings should also be evaluated for their feasibility and outcomes in diverse healthcare systems.

## 5 Conclusion

Our study demonstrates that a vaginal birth in obstetric history and an average estimated fetal weight are the most important factors for professionals to counsel patients with a breech pregnancy for VBD. We also found that the experience of professionals in assisting VBD is associated with their attitude towards VBD. Future research should focus on the centralization of breech management to improve the care for pregnant patients and their babies in breech presentation.

## Conflicts of Interest

The authors declare no conflicts of interest.

## Supporting information


**Appendix S1:** birt70011‐sup‐0001‐AppendixS1.docx.

## Data Availability

The data that support the findings of this study are available from the corresponding author upon reasonable request.
