# Supplementary material for: Professional Preferences Towards Vaginal Breech Delivery at Term: An International Discrete Choice Experiment
Source: Birth. 2025 Sep 1;53(1):174–80. doi: 10.1111/birt.70011 (PMC12894516; doi:10.1111/birt.70011)
Supplement: Supplementary file 1 — Appendix S1: birt70011‐sup‐0001‐AppendixS1.docx. [file BIRT-53-174-s001.docx]

Appendix 1: Online questionnaire

# General questions

What is your profession? - Selected Choice

How many years of clinical experience do you have?

In the last 5 years, how many vaginal breech deliveries did you assist or supervised? (1-1000)

In which country do you work?

Is upright vaginal breech delivery (all fours) at term performed at your hospital?

Would you have immediate access to an emergency operation room, if necessary?

In your hospital, is a paediatrician at standby at any time?

# Discreet choice experiment

Each participant was asked to fill in 6 random selected scenario’s as described below and answer the following question.

When it comes to giving birth, what would you advise her?

- Vaginal delivery
- Elective caesarean delivery

In the outpatient clinic you see Mrs Martin, a 22-year-old, with a singleton fetus in breech presentation after unsuccessful ECV. She is a nullipara. She is currently 37 weeks pregnant and she has an uncomplicated pregnancy. Her BMI is 23. The estimated birthweight is 2200 grams (P3). Mrs Martin has no opinion on her own.

In the outpatient clinic you see Mrs Martin, a 22-year-old, with a singleton fetus in breech presentation after unsuccessful ECV. She is a nullipara. She is currently 37 weeks pregnant and she has an uncomplicated pregnancy Her BMI is 23. The estimated birthweight is 3300 grams (P50). Mrs Martin has no opinion on her own.

In the outpatient clinic you see Mrs Martin, a 22-year-old, with a singleton fetus in breech presentation after unsuccessful ECV. She is a nullipara. She is currently 37 weeks pregnant and she has an uncomplicated pregnancy. Her BMI is 23. The estimated birthweight is 4100 grams (P97). Mrs Martin has no opinion on her own.

In the outpatient clinic you see Mrs Martin, a 22-year-old, with a singleton fetus in breech presentation after unsuccessful ECV. She is a multipara with in the obstetric history a normal vaginal birth. She is currently 37 weeks pregnant and she has uncomplicated pregnancy. Her BMI is 23. The estimated birthweight is 2200 grams (P3). Mrs Martin has no opinion on her own.

In the outpatient clinic you see Mrs Martin, a 22-year-old, with a singleton fetus in breech presentation after unsuccessful ECV. She is a multipara with in the obstetric history a normal vaginal birth. She is currently 37 weeks pregnant and she has uncomplicated pregnancy. Her BMI is 23. The estimated birthweight is 3300 grams (P50). Mrs Martin has no opinion on her own.

In the outpatient clinic you see Mrs Martin, a 22-year-old, with a singleton fetus in breech presentation after unsuccessful ECV. She is a multipara with in the obstetric history a normal vaginal birth. She is currently 37 weeks pregnant and she has uncomplicated pregnancy. Her BMI is 23. The estimated birthweight is 4100 grams (P97). Mrs Martin has no opinion on her own.

In the outpatient clinic you see Mrs Martin, a 22-year-old, with a singleton fetus in breech presentation after unsuccessful ECV. She is a multipara with in the obstetric history a caesarean for dystocia. She is currently 37 weeks pregnant and she has an uncomplicated pregnancy. Her BMI is 23. The estimated birthweight is 2200 grams (P3). Mrs Martin has no opinion on her own.

In the outpatient clinic you see Mrs Martin, a 22-year-old, with a singleton fetus in breech presentation after unsuccessful ECV. She is a multipara with in the obstetric history a caesarean for dystocia. She is currently 37 weeks pregnant and she has an uncomplicated pregnancy. Her BMI is 23. The estimated birthweight is 3300 grams (P50). Mrs Martin has no opinion on her own.

In the outpatient clinic you see Mrs Martin, a 22-year-old, with a singleton fetus in breech presentation after unsuccessful ECV. She is a multipara with in the obstetric history a caesarean for dystocia. She is currently 37 weeks pregnant and she has an uncomplicated pregnancy. Her BMI is 23. The estimated birthweight is 4100 grams (P97). Mrs Martin has no opinion on her own.

In the outpatient clinic you see Mrs Martin, a 22-year-old, with a singleton fetus in breech presentation after unsuccessful ECV. She is a nullipara. She is currently 37 weeks pregnant and she has an uncomplicated pregnancy. Her BMI is 32. The estimated birthweight is 2200 grams (P3). Mrs Martin has no opinion on her own.

In the outpatient clinic you see Mrs Martin, a 22-year-old, with a singleton fetus in breech presentation after unsuccessful ECV. She is a nullipara. She is currently 37 weeks pregnant and she has an uncomplicated pregnancy. Her BMI is 32. The estimated birthweight is 3300 grams (P50). Mrs Martin has no opinion on her own.

In the outpatient clinic you see Mrs Martin, a 22-year-old, with a singleton fetus in breech presentation after unsuccessful ECV. She is a nullipara. She is currently 37 weeks pregnant and she has an uncomplicated pregnancy. Her BMI is 32. The estimated birthweight is 4100 grams (P97). Mrs Martin has no opinion on her own.

In the outpatient clinic you see Mrs Martin, a 22-year-old, with a singleton fetus in breech presentation after unsuccessful ECV. She is a multipara with in the obstetric history a normal vaginal birth. She is currently 37 weeks pregnant and she has an uncomplicated pregnancy. Her BMI is 32. The estimated birthweight is 2200 grams (P3). Mrs Martin has no opinion on her own.

In the outpatient clinic you see Mrs Martin, a 22-year-old, with a singleton fetus in breech presentation after unsuccessful ECV. She is a multipara with in the obstetric history a normal vaginal birth. She is currently 37 weeks pregnant and she has an uncomplicated pregnancy. Her BMI is 32. The estimated birthweight is 3300 grams (P50). Mrs Martin has no opinion on her own.

In the outpatient clinic you see Mrs Martin, a 22-year-old, with a singleton fetus in breech presentation after unsuccessful ECV. She is a multipara with in the obstetric history a normal vaginal birth. She is currently 37 weeks pregnant and she has an uncomplicated pregnancy. Her BMI is 32. The estimated birthweight is 4100 grams (P97). Mrs Martin has no opinion on her own.

In the outpatient clinic you see Mrs Martin, a 22-year-old, with a singleton fetus in breech presentation after unsuccessful ECV. She is a multipara with in the obstetric history a caesarean for dystocia. She is currently 37 weeks pregnant and she has an uncomplicated pregnancy. Her BMI is 32. The estimated birthweight is 2200 grams (P3). Mrs Martin has no opinion on her own.

In the outpatient clinic you see Mrs Martin, a 22-year-old, with a singleton fetus in breech presentation after unsuccessful ECV. She is a multipara with in the obstetric history a caesarean for dystocia. She is currently 37 weeks pregnant and she has an uncomplicated pregnancy. Her BMI is 32. The estimated birthweight is 3300 grams (P50). Mrs Martin has no opinion on her own.

In the outpatient clinic you see Mrs Martin, a 22-year-old, with a singleton fetus in breech presentation after unsuccessful ECV. She is a multipara with in the obstetric history a caesarean for dystocia. She is currently 37 weeks pregnant and she has an uncomplicated pregnancy. Her BMI is 32. The estimated birthweight is 4100 grams (P97). Mrs Martin has no opinion on her own.

In the outpatient clinic you see Mrs Martin, a 32-year-old, with a singleton fetus in breech presentation after unsuccessful ECV. She is a nullipara. She is currently 37 weeks pregnant and she has an uncomplicated pregnancy. Her BMI is 23. The estimated birthweight is 2200 grams(P3). Mrs Martin has no opinion on her own.

In the outpatient clinic you see Mrs Martin, a 32-year-old, with a singleton fetus in breech presentation after unsuccessful ECV. She is a nullipara. She is currently 37 weeks pregnant and she has an uncomplicated pregnancy. Her BMI is 23. The estimated birthweight is 3300 grams (P50). Mrs Martin has no opinion on her own.

In the outpatient clinic you see Mrs Martin, a 32-year-old, with a singleton fetus in breech presentation after unsuccessful ECV. She is a nullipara. She is currently 37 weeks pregnant and she has an uncomplicated pregnancy. Her BMI is 23. The estimated birthweight is 4100 grams (P97). Mrs Martin has no opinion on her own.

In the outpatient clinic you see Mrs Martin, a 32-year-old, with a singleton fetus in breech presentation after unsuccessful ECV. She is a multipara with in the obstetric history a normal vaginal birth. She is currently 37 weeks pregnant and she has an uncomplicated pregnancy. Her BMI is 23. The estimated birthweight is 2200 grams (P3). Mrs Martin has no opinion on her own.

In the outpatient clinic you see Mrs Martin, a 32-year-old, with a singleton fetus in breech presentation after unsuccessful ECV. She is a multipara with in the obstetric history a normal vaginal birth. She is currently 37 weeks pregnant and she has an uncomplicated pregnancy. Her BMI is 23. The estimated birthweight is 3300 grams (P50). Mrs Martin has no opinion on her own.

In the outpatient clinic you see Mrs Martin, a 32-year-old, with a singleton fetus in breech presentation after unsuccessful ECV. She is a multipara with in the obstetric history a normal vaginal birth. She is currently 37 weeks pregnant and she has an uncomplicated pregnancy. Her BMI is 23. The estimated birthweight is 4100 grams (P97). Mrs Martin has no opinion on her own.

In the outpatient clinic you see Mrs Martin, a 32-year-old, with a singleton fetus in breech presentation after unsuccessful ECV. She is a multipara with in the obstetric history a caesarean for dystocia. She is currently 37 weeks pregnant and she has an uncomplicated pregnancy. Her BMI is 23. The estimated birthweight is 2200 grams (P3). Mrs Martin has no opinion on her own.

In the outpatient clinic you see Mrs Martin, a 32-year-old, with a singleton fetus in breech presentation after unsuccessful ECV. She is a multipara with in the obstetric history a caesarean for dystocia. She is currently 37 weeks pregnant and she has an uncomplicated pregnancy. Her BMI is 23. The estimated birthweight is 3300 grams (P50). Mrs Martin has no opinion on her own.

In the outpatient clinic you see Mrs Martin, a 32-year-old, with a singleton fetus in breech presentation after unsuccessful ECV. She is a multipara with in the obstetric history a caesarean for dystocia. She is currently 37 weeks pregnant and she has an uncomplicated pregnancy. Her BMI is 23. The estimated birthweight is 4100 grams (P97). Mrs Martin has no opinion on her own.

In the outpatient clinic you see Mrs Martin, a 32-year-old, with a singleton fetus in breech presentation after unsuccessful ECV. She is a nullipara. She is currently 37 weeks pregnant and she has an uncomplicated pregnancy. Her BMI is 32. The estimated birthweight is 2200 grams (P3). Mrs Martin has no opinion on her own.

In the outpatient clinic you see Mrs Martin, a 32-year-old, with a singleton fetus in breech presentation after unsuccessful ECV. She is a nullipara. She is currently 37 weeks pregnant and she has an uncomplicated pregnancy. Her BMI is 32. The estimated birthweight is 3300 grams (P50). Mrs Martin has no opinion on her own.

In the outpatient clinic you see Mrs Martin, a 32-year-old, with a singleton fetus in breech presentation after unsuccessful ECV. She is a nullipara. She is currently 37 weeks pregnant and she has an uncomplicated pregnancy. Her BMI is 32. The estimated birthweight is 4100 grams (P97). Mrs Martin has no opinion on her own.

In the outpatient clinic you see Mrs Martin, a 32-year-old, with a singleton fetus in breech presentation after unsuccessful ECV. She is a multipara with in the obstetric history a normal vaginal birth. She is currently 37 weeks pregnant and she has an uncomplicated pregnancy. Her BMI is 32. The estimated birthweight is 2200 grams (P3). Mrs Martin has no opinion on her own.

In the outpatient clinic you see Mrs Martin, a 32-year-old, with a singleton fetus in breech presentation after unsuccessful ECV. She is a multipara with in the obstetric history a normal vaginal birth. She is currently 37 weeks pregnant and she has an uncomplicated pregnancy. Her BMI is 32. The estimated birthweight is 3300 grams (P50). Mrs Martin has no opinion on her own.

In the outpatient clinic you see Mrs Martin, a 32-year-old, with a singleton fetus in breech presentation after unsuccessful ECV. She is a multipara with in the obstetric history a normal vaginal birth. She is currently 37 weeks pregnant and she has an uncomplicated pregnancy. Her BMI is 32. The estimated birthweight is 4100 grams (P97). Mrs Martin has no opinion on her own.

In the outpatient clinic you see Mrs Martin, a 32-year-old, with a singleton fetus in breech presentation after unsuccessful ECV. She is a multipara with in the obstetric history a caesarean for dystocia. She is currently 37 weeks pregnant and she has an uncomplicated pregnancy. Her BMI is 32. The estimated birthweight is 2200 grams (P3). Mrs Martin has no opinion on her own.

In the outpatient clinic you see Mrs Martin, a 32-year-old, with a singleton fetus in breech presentation after unsuccessful ECV. She is a multipara with in the obstetric history a caesarean for dystocia. She is currently 37 weeks pregnant and she has an uncomplicated pregnancy. Her BMI is 32. The estimated birthweight is 3300 grams (P50). Mrs Martin has no opinion on her own.

In the outpatient clinic you see Mrs Martin, a 32-year-old, with a singleton fetus in breech presentation after unsuccessful ECV. She is a multipara with in the obstetric history a caesarean for dystocia. She is currently 37 weeks pregnant and she has an uncomplicated pregnancy. Her BMI is 32. The estimated birthweight is 4100 grams (P97). Mrs Martin has no opinion on her own.

In the outpatient clinic you see Mrs Martin, a 42-year-old, with a singleton fetus in breech presentation after unsuccessful ECV. She is a nullipara. She is currently 37 weeks pregnant and she has an uncomplicated pregnancy. Her BMI is 23. The estimated birthweight is 2200 grams (P3). Mrs Martin has no opinion on her own.

In the outpatient clinic you see Mrs Martin, a 42-year-old, with a singleton fetus in breech presentation after unsuccessful ECV. She is a nullipara. She is currently 37 weeks pregnant and she has an uncomplicated pregnancy. Her BMI is 23. The estimated birthweight is 3300 grams (P50). Mrs Martin has no opinion on her own.

In the outpatient clinic you see Mrs Martin, a 42-year-old, with a singleton fetus in breech presentation after unsuccessful ECV. She is a nullipara. She is currently 37 weeks pregnant and she has an uncomplicated pregnancy. Her BMI is 23. The estimated birthweight is 4100 grams (P97). Mrs Martin has no opinion on her own.

In the outpatient clinic you see Mrs Martin, a 42-year-old, with a singleton fetus in breech presentation after unsuccessful ECV. She is a multipara with in the obstetric history a normal vaginal birth. She is currently 37 weeks pregnant and she has an uncomplicated pregnancy. Her BMI is 23. The estimated birthweight is 2200 grams (P3). Mrs Martin has no opinion on her own.

In the outpatient clinic you see Mrs Martin, a 42-year-old, with a singleton fetus in breech presentation after unsuccessful ECV. She is a multipara with in the obstetric history a normal vaginal birth. She is currently 37 weeks pregnant and she has an uncomplicated pregnancy. Her BMI is 23. The estimated birthweight is 3300 grams (P50). Mrs Martin has no opinion on her own.

In the outpatient clinic you see Mrs Martin, a 42-year-old, with a singleton fetus in breech presentation after unsuccessful ECV. She is a multipara with in the obstetric history a normal vaginal birth. She is currently 37 weeks pregnant and she has an uncomplicated pregnancy. Her BMI is 23. The estimated birthweight is 4100 grams (P97). Mrs Martin has no opinion on her own.

In the outpatient clinic you see Mrs Martin, a 42-year-old, with a singleton fetus in breech presentation after unsuccessful ECV. She is a multipara with in the obstetric history a caesarean for dystocia. She is currently 37 weeks pregnant and she has an uncomplicated pregnancy. Her BMI is 23. The estimated birthweight is 2200 grams (P3). Mrs Martin has no opinion on her own.

In the outpatient clinic you see Mrs Martin, a 42-year-old, with a singleton fetus in breech presentation after unsuccessful ECV. She is a multipara with in the obstetric history a caesarean for dystocia. She is currently 37 weeks pregnant and she has an uncomplicated pregnancy. Her BMI is 23. The estimated birthweight is 3300 grams (P50). Mrs Martin has no opinion on her own.

In the outpatient clinic you see Mrs Martin, a 42-year-old, with a singleton fetus in breech presentation after unsuccessful ECV. She is a multipara with in the obstetric history a caesarean for dystocia. She is currently 37 weeks pregnant and she has an uncomplicated pregnancy. Her BMI is 23. The estimated birthweight is 4100 grams (P97). Mrs Martin has no opinion on her own.

In the outpatient clinic you see Mrs Martin, a 42-year-old, with a singleton fetus in breech presentation after unsuccessful ECV. She is a nullipara. She is currently 37 weeks pregnant and she has an uncomplicated pregnancy. Her BMI is 32. The estimated birthweight is 2200 grams (P3). Mrs Martin has no opinion on her own.

In the outpatient clinic you see Mrs Martin, a 42-year-old, with a singleton fetus in breech presentation after unsuccessful ECV. She is a nullipara. She is currently 37 weeks pregnant and she has an uncomplicated pregnancy. Her BMI is 32. The estimated birthweight is 3300 grams (P50). Mrs Martin has no opinion on her own.

In the outpatient clinic you see Mrs Martin, a 42-year-old, with a singleton fetus in breech presentation after unsuccessful ECV. She is a nullipara. She is currently 37 weeks pregnant and she has an uncomplicated pregnancy. Her BMI is 32. The estimated birthweight is 4100 grams (P97). Mrs Martin has no opinion on her own.

In the outpatient clinic you see Mrs Martin, a 42-year-old, with a singleton fetus in breech presentation after unsuccessful ECV. She is a multipara with in the obstetric history a normal vaginal birth. She is currently 37 weeks pregnant and she has an uncomplicated pregnancy. Her BMI is 32. The estimated birthweight is 2200 grams (P3). Mrs Martin has no opinion on her own.

In the outpatient clinic you see Mrs Martin, a 42-year-old, with a singleton fetus in breech presentation after unsuccessful ECV. She is a multipara with in the obstetric history a normal vaginal birth. She is currently 37 weeks pregnant and she has an uncomplicated pregnancy. Her BMI is 32. The estimated birthweight is 3300 grams (P50). Mrs Martin has no opinion on her own.

In the outpatient clinic you see Mrs Martin, a 42-year-old, with a singleton fetus in breech presentation after unsuccessful ECV. She is a multipara with in the obstetric history a normal vaginal birth. She is currently 37 weeks pregnant and she has an uncomplicated pregnancy. Her BMI is 32. The estimated birthweight is 4100 grams (P97). Mrs Martin has no opinion on her own.

In the outpatient clinic you see Mrs Martin, a 42-year-old, with a singleton fetus in breech presentation after unsuccessful ECV. She is a multipara with in the obstetric history a caesarean for dystocia. She is currently 37 weeks pregnant and she has an uncomplicated pregnancy. Her BMI is 32. The estimated birthweight is 2200 grams (P3). Mrs Martin has no opinion on her own.

In the outpatient clinic you see Mrs Martin, a 42-year-old, with a singleton fetus in breech presentation after unsuccessful ECV. She is a multipara with in the obstetric history a caesarean for dystocia. She is currently 37 weeks pregnant and she has an uncomplicated pregnancy. Her BMI is 32. The estimated birthweight is 3300 grams (P50). Mrs Martin has no opinion on her own.

In the outpatient clinic you see Mrs Martin, a 42-year-old, with a singleton fetus in breech presentation after unsuccessful ECV. She is a multipara with in the obstetric history a caesarean for dystocia. She is currently 37 weeks pregnant and she has an uncomplicated pregnancy. Her BMI is 32. The estimated birthweight is 4100 grams (P97). Mrs Martin has no opinion on her own.

# Intrapartum management

**Case 1:** Mrs Martin, a 32-year-old a nullipara, has a singleton fetus in frank breech presentation and is 39 weeks pregnant. Mrs Martin has a BMI of 27 and her medical history is unremarkable. She opted for vaginal delivery after shared decision-making. The estimated birthweight is 3300 grams (P50), and CTG registration demonstrates a reactive fetal condition. Mrs Martin has mild preeclampsia and according to the national guidelines, termination of pregnancy is recommended.

Question 1: Would you consider induction?

- Yes
- Yes, only with a favourable cervix, otherwise an elective caesarean is advised
- No, an elective caesarean delivery is advised

**Case 2:** Mrs Martin, a 32-year-old a nullipara, has a singleton fetus in frank breech

presentation and is 39 weeks pregnant. Mrs Martin has a BMI of 27 and her medical history is unremarkable. She opted for vaginal delivery after shared decision-making. The estimated birthweight is 3300 grams (P50), and CTG registration demonstrates a reactive fetal condition. Mrs Martin has an uncomplicated pregnancy and went into spontaneous labour.

Question 2.1: Would you want to use CTG registration during the whole delivery?

- Yes
- No

Question 2.2: Would you allow pain relief of all kinds? (e.g. remifentanil, epidural)

- Yes
- No

Question 2.3: Would you allow the patient to change her position during delivery?

- Yes
- No

Question 2.4: Would you consider augmentation for a lack of progression due to inadequate contractions? (yes = Oxytocin intravenously will be administered according to our local protocol / No = caesarean delivery is advised)

- Yes
- No

Question 2.5: Would you consider augmentation for a lack of progression in active phase of labour? (yes = Oxytocin intravenously will be administered according to our local protocol / No = caesarean delivery is advised)

**Case 3:** Mrs Martin, a 32-year-old a nullipara, has an uncomplicated singleton breech pregnancy in frank position and is 39 weeks pregnant. Mrs Martin has a BMI of 27 and her medical history is unremarkable. Previously in the outpatient clinic, she opted for vaginal delivery after shared decision-making. The estimated birthweight is 3300 grams (P50). Now Mrs Martin is in the active phase of labour, without intravenous oxytocin or pain relief.

Question 3.1: She is in the pushing phase for 30 minutes with adequate contractions (in frequency and in progression). The breech is at station 0/ Hodge 3. For 15 minutes, there is an abnormal CTG according to FIGO classification, indicating fetal distress.

What would you consider? - Selected Choice

- Emergency caesarean delivery
- Continue active pushing
- Breech extraction
- Other, please describe...

Question 3.2: She is in the pushing phase for 45 minutes with adequate contractions (in frequency and in progression). The breech is at station 0/ Hodge 3. For 15 minutes, there is an abnormal CTG according to FIGO classification, indicating fetal distress.

What would you consider? - Selected Choice

- Emergency caesarean delivery
- Continue active pushing
- Breech extraction
- Other, please describe...

Question 3.3: She is in the pushing phase for 30 minutes with adequate contractions (in frequency and in progression). The breech is crowning. For 15 minutes, there is an abnormal CTG according to FIGO classification, indicating fetal distress.

What would you consider? - Selected Choice

- Emergency caesarean delivery
- Continue active pushing
- Breech extraction
- Other, please describe...

Question 3.4: She is in the pushing phase for 45 minutes with adequate contractions (in frequency and in progression). The breech is crowning. For 30 minutes, there is an abnormal CTG according to FIGO classification, indicating fetal distress.

What would you consider? - Selected Choice

- Emergency caesarean delivery
- Continue active pushing
- Breech extraction
- Other, please describe...
